# Maternal *Faecalibacterium* pathobionts and low-fiber diets synergize to impact offspring health: implications for atopic dermatitis

**DOI:** 10.1186/s40168-025-02194-8

**Published:** 2025-08-29

**Authors:** Dongju Lee, Jongwook Park, Song-Yi Park, Junghyun Hwang, Sewon Kim, Sun-Ho Kee, Heenam Stanley Kim

**Affiliations:** 1 Division of Biosystems & Biomedical Sciences, College of Health Sciences, Korea University, 145 Anam-Ro, Seongbuk-Gu, Seoul, 02841 Korea; 2 Department of Microbiology, College of Medicine, Korea University, 145 Anam-Ro, Seongbuk-Gu, Seoul, 02841 Korea

**Keywords:** *Faecalibacterium*, Pathobiont, Gut microbiome, Atopic dermatitis, Dietary fiber, Systemic conditions

## Abstract

**Background:**

The incidence of atopic dermatitis (AD) has increased globally in recent decades. A recent study identified enrichment of *Faecalibacterium* subspecies in young AD patients, implicating these gut bacteria in disease pathogenesis. This was unexpected, as *Faecalibacterium* is widely recognized as one of the most beneficial bacteria in the human gut.

**Results:**

We tested the bacteria in female mice and observed their effect on the gut microbiome and overall health, which subsequently influenced the health of their offspring. These effects were markedly exacerbated when female mice were fed a low-fiber diet, leading to heightened systemic inflammation, skin damage, and hair loss in their offspring. Offspring of female mice receiving a low-fiber diet without pathobiont administration exhibited reduced symptom severity, which was further mitigated bythe administration of the beneficial strain A2-165.

**Conclusions:**

These findings provide compelling evidence that maternal*Faecalibacterium* pathobionts play a critical role in the development of systemic conditions in offspring, offering valuable insights into the etiology of AD. Furthermore, the synergistic effect of gut microbiota dysbiosis and low fiber intake highlights the potential impact of modern dietary trends on the rising prevalence of AD and other chronic conditions.

Video Abstract

**Supplementary Information:**

The online version contains supplementary material available at 10.1186/s40168-025-02194-8.

## Background

The prevalence of atopic dermatitis (AD) has increased globally, particularly in urban regions of developed nations, affecting approximately 30% of the pediatric population [1–3]. Although AD is not life-threatening, it can significantly reduce the quality of life [1, 3–5]. Onset typically begins between 3 and 6 months of age, with the most affected children showing symptoms within the first 12 months [6], suggesting that maternal factors may contribute to disease development. Over the past few decades, understanding of the pathophysiology of AD has significantly progressed. The key advances include the recognition of the complex interplay between a compromised epidermal barrier, an abnormal skin microbiome, and type-2 immune dysregulation in skin tissues, which drives inflammatory symptoms [7, 8]. Moreover, a disrupted skin microbiome, characterized by significantly reduced species diversity and the dominance of *Staphylococcus aureus* as both a colonizer and pathogen, has been identified as a key contributor to AD pathogenesis [9–11]. Increasing evidence suggests that AD is not merely a dermatological condition but a systemic disease. This finding is supported by its frequent association with the later development of atopic disorders, including asthma, allergic rhinitis, and food allergies [1, 3–5]. Furthermore, AD has been linked to various non-atopic conditions including mental health disorders, such as depression, anxiety, and attention-deficit hyperactivity disorder, and inflammatory diseases, such as inflammatory bowel disease (IBD) [12–14]. AD is part of a broad spectrum of chronic conditions, including autoimmune diseases, metabolic conditions, and inflammatory bowel diseases, which are associated with disturbances in the gut microbiota in modern populations [8, 15, 16]. Although heritability has been reported in some AD studies [17, 18], these findings may result from disrupted gut microbiomes being passed down through generations [15].

The reduced abundance of *Faecalibacterium prausnitzii* has been associated with various conditions including Crohn’s disease [19], ulcerative colitis [20], and metabolic disorders [21], highlighting its role as a major beneficial gut bacterium. *F. prausnitzii* supports gut homeostasis in various ways, most notably through the production of butyric acid, which is a crucial metabolite in gut health [22, 23]. However, a significant increase in *F. prausnitzii* related to strain L2-6 has been identified in the gut of young patients with AD, suggesting a potential role of these bacteria in AD pathogenesis [24]. This finding aligns with that of a study on pediatric Crohn’s disease demonstrating that L2-6-like strains were more abundant than beneficial A2-165-like strains in patients [25]. Similarly, South Indian children with obesity had significantly higher *F. prausnitzii* levels than their counterparts without obesity [26]. Thus, *F. prausnitzii* pathobionts may be involved in various conditions, particularly in children. Phylogenetic analyses of *F. prausnitzii* strains using comparative genomics support this observation, revealing that strain L2-6 is distinct from health-benefiting strains such as A2-165 [27].

Despite 16S rRNA gene sequences showing more than 97% matches, *F. prausnitzii* has been classified into several distinct species based on genome-based phenotypic and chemotaxonomic criteria. Based on this classification, strain L2-6 was reclassified as *Faecalibacterium longum*, whereas strain A2-165 was designated as *Faecalibacterium duncaniae* [28, 29].

In this study, we used a mouse model to investigate the potential role of *Faecalibacterium* strains isolated from patients with AD in the development of systemic conditions. Administration of these strains disrupted gut homeostasis in female mice and adversely affected their offspring. These effects were exacerbated when female mice were fed a low-fiber diet, leading to severe systemic symptoms in their offspring including hair loss. This study highlights the potential causal role of maternal *Faecalibacterium* pathobionts in offspring and provides valuable insights into the etiology of AD and other chronic conditions in humans.

## Methods

### Faecalibacterium strains and cultures

*F. duncaniae* A2-165 (DSM 17677) was obtained from the German Collection of Microorganisms and Cell Cultures (Braunschweig, Germany). Additional *Faecalibacterium* strains were isolated from human fecal samples, as described in our earlier publication [24]. All bacterial cultures were prepared in an anaerobic chamber (Coy Laboratory Products, Grass Lake, Michigan, USA), maintaining anaerobic conditions (N₂; 85%, CO₂; 10%, H₂; 5%). Human fecal samples were mixed with anaerobic reinforced clostridial medium (RCM) broth (BD Difco™; Becton Drive, Franklin Lakes, NJ, USA) and plated on RCM agar plates. Plates were incubated at 37℃ for 2–3 days until colonies became visible. The colonies were then re-streaked on fresh RCM agar plates to obtain pure single colonies, ensuring the isolation of individual strains. Each colony was subsequently inoculated into 3 mL of RCM broth and incubated overnight at 37℃. A 1-mL portion of the inoculum was preserved as a glycerol stock, and the remainder was used for bacterial genomic DNA extraction using the Wizard Genomic DNA Purification Kit (Promega, Madison, WI, USA). Isolates were identified via PCR amplification using the 7 F (5’-AGAGTTTGATYMTGGCTCAG-3’) and 1510R (5’-ACGGYTACC TTGTTACGACTT-3’) primers, with 16S rRNA gene sequencing using a 3730 DNA analyzer (Applied Biosystems, Foster City, CA, USA) by Macrogen Inc. (Seoul, Korea).

### Scanning electron microscopy (SEM) of Faecalibacterium strains

Each *Faecalibacterium* strain was cultured overnight and then inoculated into 20 ml of fresh RCM broth at a 1:100 ratio, incubating at 37 ℃ until an OD_600_ of 0.7–0.8 was reached. Subsequently, 1 mL of the log-phase bacterial culture was pelleted using centrifugation at 1000 × *g* for 10 min. After discarding the supernatant, the cells were washed with phosphate-buffered saline (PBS) and fixed for 2 h in 2.5% glutaraldehyde in 0.1 M phosphate buffer (pH 7.4). The cells were then washed twice with the same buffer for 20 min each, followed by a 2-h post-fixation with 2% osmium tetroxide. After a brief rinse with distilled water, the cells were dehydrated in an ethanol series and freeze-dried using a vacuum freeze-drier (ES-2030; Hitachi, Tokyo, Japan). The prepared samples were mounted on a stub, coated with platinum using an ion-sputter (Hitachi E-1045), and visualized using SEM (Hitachi S-4700).

### PBMC assays

Human PBMCs were obtained from Komabiotech (PBMNC100C; Seoul, Korea), washed with RPMI-1640 medium (R8758; Sigma Aldrich), and adjusted to a concentration of 2 × 10^6^ cells/mL in RPMI-1640 supplemented with 10% FBS (35–015-CV; Corning, Glendale, AZ, USA) and 150 µg/mL gentamicin. The cells were seeded at 1 ml per well in 24-well tissue culture plates and equilibrated at 37℃ in an atmosphere of 5% CO_2_. After 24 h, 20 µL of a bacterial suspension (10^8^ CFU/mL) was added to each well, with RCM broth serving as the negative control. Following 24 h of stimulation, the samples were collected, transferred to tubes, and centrifuged at 12,000 × *g* for 3 min at 4℃ to pellet the cells. Supernatants were carefully collected and stored at − 20℃ until further analysis. The IL-10 concentration in the supernatants was measured using a Human IL-10 ELISA kit (K0331123; Komabiotech, Seoul, Korea).

### Development of a mouse model to assess the effects of Faecalibacterium strains on the host

Male and female specific pathogen-free (SPF) C57BL/6N mice, aged 6–7 weeks, were obtained from Koatech (Gyunggi-do, Korea) and housed under controlled conditions at a temperature of 21 ± 2℃, relative humidity of 45% ± 5%, and a 12:12-h light–dark cycle. The mice were acclimated to these conditions for 1 week before being used in the experiments. All animal procedures were conducted according to the guidelines of the Central Laboratory Animal Research Center of Korea University (Seoul, Korea).

**Fig. 1:**
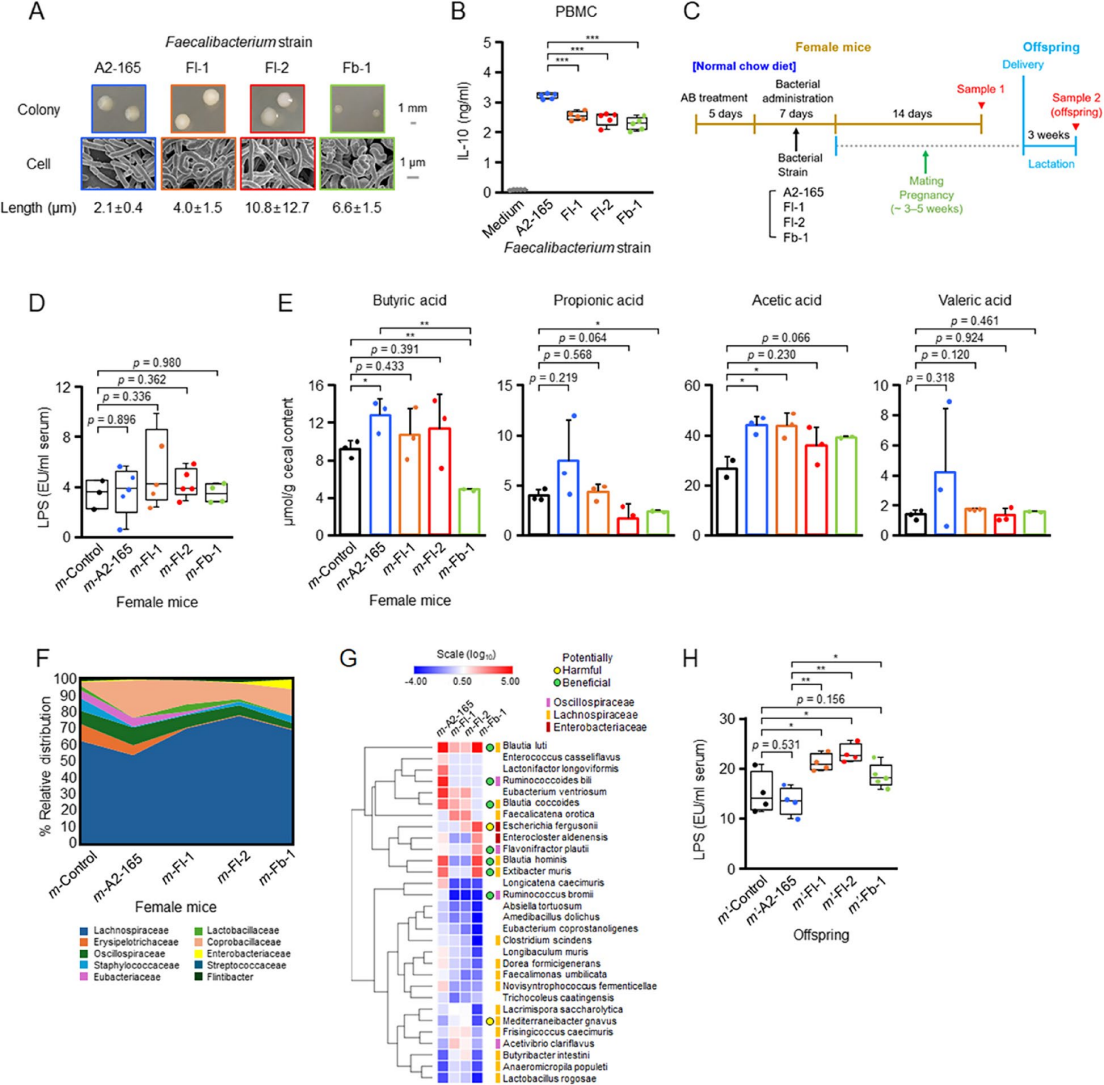
Effects of *Faecalibacterium* strains on female mice and their offspring. **A***Faecalibacterium* isolates obtained from patients with atopic dermatitis (AD). Colonies on growth plates and scanning electron microscope (SEM) images of cells are shown. **B** IL-10 secretion from PBMCs in response to *Faecalibacterium* strains. **C** Mouse model used for testing *Faecalibacterium* strains. Sample 1 was collected from female mice that did not undergo pregnancy, whereas sample 2 was obtained from offspring before weaning. **D** Serum LPS levels in groups of female mice (*n* = 5 per group). **E** Cecal short-chain fatty acid (SCFA) levels in groups of female mice. **F** Gut microbiota profiles of groups of female mice. **G** Heatmap of hierarchical clustering showing the relative abundance of key bacterial species among the mouse groups on day 14 post-administration. The log_10_ ratios of bacterial species in *m-*A2-165, *m-*Fl-1, *m-*Fl-2, or *m-*Fb-1 relative to those in *m-*Control are shown. Potentially harmful or beneficial bacteria, as well as three key families to which these bacterial species belong, are color-coded for distinction. **H** Serum LPS levels in offspring. Students *t*-test; *p < 0.05; **p < 0.01; and ***p < 0.001. Statistics are presented for results that met the specified *p* value thresholds and for others that were noteworthy

The overall schematic is displayed in Fig. 1C. Prior to the administration of bacterial suspensions, mice were administered ampicillin (1 g/L; Sigma Aldrich), vancomycin (500 mg/L; Sigma Aldrich), and neomycin (1 g/L; Sigma Aldrich) in drinking water ad libitum for 5 days to minimize the resident gut flora. Mice were then administered bacterial suspensions from overnight cultures (10^8^–10^9^ CFU in 0.25 mL) or an equivalent volume of RCM broth as a control via intragastric gavage for 7 days. Approximately 10 min before each gavage, the mice received 0.1 mL of 0.2 M sodium bicarbonate to neutralize stomach acids and improve bacterial survival [30]. Following the 7-day administration period, the mice were divided into two groups. One group was assessed on the 14th day after bacterial administration (sample 1). In the other group, male mice were introduced into the cages of female mice for mating at a 1:2 ratio. Female mice were weighed every 3 days to monitor for signs of pregnancy, and those showing significant weight increases were separated from the group. These mice were then maintained through pregnancy, delivery, and lactation, with the health of their offspring evaluated at 3 weeks of age.

### Normal and a low-fiber diet

The normal diet consisted of standard rodent chow (Altromin 1314; Altromin, Lage, Germany), which typically contains high levels of dietary fiber, comprising approximately 20% of the diet. The low-fiber diet was a modified version of Harlan TD.088110 customized by DooYeol Biotech (Seoul, Korea), with the dietary fiber content reduced to 8%.

### Animal sample processing

Female mice and 3-week-old offspring were anesthetized with isoflurane, and the blood was drawn from the postcaval vein using a 1-mL syringe with a 25-G needle. The blood was transferred to a Microtainer SST tube (BD) and gently mixed by inverting 3–4 times. Serum was separated by centrifugation at 12,000 × *g* at 4 ℃ for 3 min and stored at − 20℃ until analysis. Serum immune factors were measured following the manufacturer’s instructions, using the Pierce™ LAL Chromogenic Endotoxin Quantification kit (A39553; Thermo Fisher), Mouse IL-10 ELISA kit (K0331213; Komabiotech), IL-17 Mouse ELISA kit (BMS6001; Invitrogen), IL-23 Mouse ELISA kit (BMS6017; Invitrogen), Mouse Zonulin ELISA kit (MBS748504; MyBioSource, San Diego, USA), Mouse Calprotectin ELISA kit (MBS7606640; MyBioSource), and Mouse CRP ELISA Kit (EM20RB; Invitrogen).

Following blood collection, the mice were sacrificed in a CO_2_ chamber, and their intestines were promptly removed and rinsed with PBS. The intestines were stored at − 80℃ for later analysis or fixed in either neutral buffered 10% formalin or methanol − Carnoy’s solution (dry methanol to chloroform to glacial acetic acid, 60:30:10 (v/v/v)). For methanol − Carnoy’s fixation, the intestines were immersed in the solution for 2 h, transferred to fresh methanol − Carnoy’s solution overnight, and then stored at 4℃. The skin from the lower back of the offspring was collected after intestinal extraction and fixed in 10% formalin. Fixed samples were embedded in paraffin, and thin sections were stained with hematoxylin and eosin (H&E) or periodic acid-schiff/alcian blue (PAS/AB).

### SCFA analysis

To assess SCFA production by, or influenced by, *Faecalibacterium* strains, overnight cultures were centrifuged at 3500 rpm for 10 min at 4℃. The supernatant was then filtered through a 0.45-µm syringe filter (Minisart, Sartorius, Germany). A 200-µL aliquot of the filtered supernatant was transferred to a new tube, and its weight was recorded. To this, 800 µL of 80% methanol and 5 µL of 2-ethylbutyric acid stock (13 mg/mL in 80% methanol) as an internal standard were added, and the mixture was vortexed thoroughly. For SCFA analysis of mouse cecal content, approximately 100 mg of cecal material was placed into a 2.0-mL screw-cap tube containing 500 mg of 0.1-mm zirconia-silica beads (Biospec Products, Bartlesville, OK, USA), and its weight was recorded. One milliliter of 80% methanol and 5 µL of 2-ethylbutyric acid stock solution were added. The mixture was homogenized using a Mini-Beadbeater-24 (Biospec Products) at 2400 rpm for 4 min, followed by a 4-min cooling period; this cycle was repeated three times. After homogenization, the mixture was centrifuged at 14,000 rpm for 5 min at 4℃, and the supernatant was filtered through a 0.45-µm syringe filter. SCFAs were analyzed using a DB-FFAP column (50 m × 0.32 mm, 0.50 µm; J&W Scientific, Folsom, CA, USA) and an Agilent 7890 A gas chromatograph (Agilent Technologies, Santa Clara, CA, USA) equipped with a flame ionization detector.

### H&E staining of the intestine and skin tissues

H&E staining was performed on the intestinal and skin tissues. Briefly, tissues were fixed in 4% buffered formalin, processed routinely, embedded in paraffin, and sectioned into 4-μm slices. Sections were dewaxed in xylene, stained with H&E (Vector Laboratories, Burlingame, CA, USA), rehydrated in graded alcohol solution, and cleared in xylene. Finally, the sections were mounted using Paramount (Fisher Scientific, NH, USA) and examined under an optical microscope (BX51; Olympus Corporation, Tokyo, Japan).

### Immunohistochemistry on gut mucosa and skin tissues

Immunohistochemistry was used to detect the expression of E-cadherin, ZO-1, and MUC2 in the intestinal tissue, and ZO-1, claudin-1, and claudin-7 in the skin tissue. Tissue sections were dewaxed in xylene, rehydrated in graded alcohol solution, and treated with 3% H_2_O_2_ at room temperature for 10 min to deactivate endogenous peroxidase. The antigen was retrieved by microwave heating at 400 W for 5 min, which was repeated three times. To block nonspecific binding, the sections were incubated with 5% normal goat serum (diluted in PBS) at room temperature for 1 h. Primary antibodies—anti-E-cadherin (1:200, BD transduction, NJ, USA), anti-ZO-1 (1:100, Invitrogen, MA, USA), anti-MUC2 (1:100, Novus, MS, USA), and anti-claudin-1 and −7 (1:100, Invitrogen, MA, USA)—were applied, and sections were incubated overnight at 4°C. Sections were then incubated with goat anti-rabbit IgG and horseradish peroxidase-conjugated secondary antibodies (1:100; Vector Laboratories, Burlingame, CA, USA) for 1 h at room temperature. After these PBS washes, the avidin–biotin–peroxidase complex was applied, and detection was performed with 3,3′-diaminobenzidine (Vector Laboratories, Burlingame, CA, USA). Sections were counterstained with hematoxylin, dehydrated using xylene and graded alcohol solution, and mounted. Images were captured using an optical microscope (BX51; Olympus Corporation, Tokyo, Japan).

### Quantification of MUC2 production in histological images

To quantify MUC2 production, PAS-positive regions in histological images were measured at three randomly selected sites per section using ImageJ [31]. Measurements for each group were normalized to the levels observed in the *m*ʹ-Control_ND group and expressed as percentage values.

### 16S rRNA gene amplicon sequencing of cecal microbiota samples

To analyze the cecal microbiota of female mice, as well as mother and offspring pairs, genomic DNA was extracted from cecal content using the QIAamp PowerFecal Pro DNA kit (Qiagen, Hilden, Germany). The extraction process included an agitation step performed using a Mini-Beadbeater-24 (Biospec Products) at 3800 rpm for 5 min, followed by a 5-min chilling period, and an additional 5 min of agitation. DNA was quantified using the Quant-iT PicoGreen assay (Invitrogen).

Amplicon libraries for the three groups of mice (female, mother, and offspring) were prepared separately by Macrogen Inc. (Seoul, Korea) following Illumina 16S Metagenomic Sequencing Library protocols, targeting the V3 and V4 regions of the 16S rRNA gene. Briefly, 5 ng of input gDNA was subjected to PCR amplification in a reaction containing 5 × buffer, 1 mM dNTP mix, 500 nM of each universal forward and reverse PCR primer, and Herculase II fusion DNA polymerase (Agilent Technologies, Santa Clara, CA, USA). The initial PCR cycle included heat activation at 95 °C for 3 min, followed by 25 cycles of denaturation at 95 °C for 30 s, annealing at 55 °C for 30 s, extension at 72 °C for 30 s, and a final extension at 72 °C for 5 min. The primer sequences with Illumina adapter overhangs used in the first amplifications were as follows: V3-F: 5′-TCGTCGGCAGCGTCAGATGTGTATAAGAGACAGCCTACGGGNGGCWGCAG-3′, V4-R: 5′-GTCTCGTGGGCTCGGAGATGTGTATAAGAGACAGGACTACHVGGGTATCTAATCC-3′. The first PCR product was purified using AMPure beads (Agencourt Bioscience, Beverly, MA). Next, 2 μL of the purified PCR product underwent a second PCR amplification for library construction, incorporating indexing primers from the Nextera XT Indexed Primer kit (Illumina, San Diego, USA). The second PCR was performed under the same conditions as the first, but with only 10 cycles. The resulting product was purified using the AMPure beads. The final library was quantified using qPCR, following the KAPA Library Quantification protocol for Illumina platforms, and the quality was assessed using TapeStation D1000 ScreenTape (Agilent Technologies, Santa Clara, CA, USA). Paired-end sequencing (2 × 300 bp) was then performed on the MiSeq™ platform (Illumina, San Diego, USA) by Macrogen Inc. (Seoul, Korea).

Following sequencing on the Illumina MiSeq platform, raw data were demultiplexed using index sequences to generate paired-end FASTQ files for each sample. The forward and reverse sequences of each sample were deposited in the National Center for Biotechnology Information (NCBI) under BioProject accession numbers PRJNA1227222 (female), PRJNA1227227 (mother), and PRJNA1227231 (offspring). Adapter and primer sequences were removed using Cutadapt (v.3.2) within QIIME (v.1.9). Quality filtering, error correction, noise and chimera sequence removal, and amplicon sequence variant (ASV) formation were performed using the DADA2 packagee (v1.18.0) in the R environment (v.4.0.3), excluding ASVs shorter than 350 bp. Taxonomics were assigned to each ASV using MegaBLAST searches against the NCBI 16S Microbial Database, with taxonomic identities selected based on the highest sequence identity, requiring a minimum of 85% identity and coverage. ASVs with < 85% identity were not assigned a taxonomy. To present the data, log_10_ ratios of bacterial species were calculated by comparing mice that received bacterial administration to control mice that did not. For bacterial species with no detectable reads, a single read was assigned to enable ratio calculations. Bacterial species showing differential abundance across mouse groups were then identified. Heatmaps with hierarchical clustering of the selected species were generated using the Morpheus software (https://software.broadinstitute.org/morpheus/).

### Quantification of the hair-loss region on the body

The anesthetized offspring were placed on a white sheet of paper and photographed. Each image was converted into 8-bit format with appropriate threshold adjustments. A specific region was defined extending from the area between the ears to the buttocks, excluding the face, ears, paws, and tails. Within this defined area, the percentage of hair loss relative to the total area was calculated. This analysis was performed using the ImageJ software (https://github.com/imagej/ImageJ).

## Results

### Effects of Faecalibacterium strains on female mice and their offspring

We previously observed that L2-6-related *F. prausnitzii*, now reclassified as *F. longum* [28], was significantly increased in young patients with AD [24]. To further investigate the impact of these bacteria on the host, we isolated strains from the fecal samples of patients with AD and selected two strains, designated Fl-1 and Fl-2, for further analysis. Additionally, we included a strain identified as *Faecalibacterium butyricigenerans*, labeled Fb-1 (Fig. 1A).

These strains displayed distinct morphologies at both the colony and cellular levels, with cells notably longer and curlier than those of the beneficial strain *F. duncaniae* A2-165 (Fig. 1A). Moreover, their interaction with human peripheral blood mononuclear cells (PBMCs) differed from that of strain A2-165, showing a significantly lower induction of the anti-inflammatory cytokine IL-10 (Fig. 1B).

To investigate the potential effects of these *Faecalibacterium* isolates in mice, we orally administered each isolate to 8-week-old female C57BL/6N mice via gavage (Fig. 1C). The mice were then divided into two groups; one group was assessed on the 14th day post-bacterial administration (sample 1), whereas the other group was assessed after pregnancy, delivery, and lactation, and the condition of their offspring examined at 3 weeks of age (sample 2) (Fig. 1C). When serum lipopolysaccharide (LPS) levels were measured in sample 1, mice administered strains Fl-1 or Fl-2 (referred to as *m*-Fl-1 and *m*-Fl-2, respectively) consistently showed moderately elevated levels—particularly *m*-Fl-1—compared to those given only the growth medium (*m*-Control). However, these differences did not reach statistical significance (Fig. 1D). Additional tests in sample 1, including serum levels of C-reactive protein (CRP), calprotectin, and IL-17A, revealed no significant changes compared to *m*-Control (Additional file 1: Fig. S1). The cecal levels of major short-chain fatty acids (SCFAs)—butyric acid, propionic acid, acetic acid, and valeric acid—appeared to be differentially affected by the various *Faecalibacterium* strains (Fig. 1E). For instance, mice administered strains Fl-1 or Fl-2 (*m*-Fl-1 and *m*-Fl-2) showed modest increases in butyric and acetic acid levels, whereas propionic and valeric acid levels were slightly reduced or comparable to those in the *m*-Control group. Similarly, the *m*-Fb-1 group tended to exhibit lower levels of butyric and propionic acids relative to controls. Although these changes represented relatively weak trends overall, mice colonized with the beneficial strain *m*-A2-165 consistently showed elevated levels across all four SCFAs, often surpassing the threshold for statistical significance (Fig. 1E). Decreased butyric and propionic acid levels have been reported in the fecal samples of patients with AD [24]. This observation aligns with the reduction in propionic acid levels in *m*-Fl-1 and *m*-Fl-2 but not with the trends observed for butyric acid. A microbiome with elevated valeric acid levels is considered healthy because these levels play a role in effectively controlling *Clostridioides difficile* infection [32]. By the 14th day post-administration, the bacterial strains administered to mice in sample 1 were not detectable in the cecal contents via DNA sequencing. However, significant global changes in the cecal microbiota structure were observed in mice treated with *Faecalibacterium* strains (Fig. 1F), accompanied by substantial shifts in the relative abundance of potentially influential bacterial species (Fig. 1G). Most of the differentially altered species across the mouse groups belonged to the families Lachnospiraceae and Oscillospiraceae, the latter of which includes *Faecalibacterium* (Fig. 1G). Altogether, these families represent butyrate producers that reside in the mucin layer, particularly within the crypts and transverse folds of the large intestine [33, 34]. This localization may explain why these family members were the most impacted by the introduction of *Faecalibacterium* strains as co-residents of these niches. The alterations followed several patterns as follows: (1) a minimal increase or decrease in potentially beneficial species in *m*-Fl-1, *m*-Fl-2, or *m*-Fb-1 than in *m*-A2-165; (2) an increase in potentially harmful species in *m*-Fl-1, *m*-Fl-2, or *m*-Fb-1 than in *m*-A2-165; and (3) a minimal decrease in potentially harmful species in *m*-Fl-1, *m*-Fl-2, or *m*-Fb-1than in *m*-A2-165 (Fig. 1G). The first pattern was observed in *Blautia luti* [35], *Blautia coccoides* [35], *Blautia hominis* [35], *Ruminococcoides bili* [36], and *Extibacter muris* [37] (Fig. 1G). The second pattern was noted in *Escherichia fergusonii* [38], and the third was evident in *Mediterraneibacter gnavus* [39] (Fig. 1G). Additional species that exhibit similar patterns may have comparable effects in each mouse group. The offspring of *m*-Fl-1, *m*-Fl-2, and *m*-Fb-1 (designated as *m*ʹ-Fl-1, *m*ʹ-Fl-2, and *m*ʹ-Fb-1, respectively) exhibited significantly elevated LPS levels than the offspring of *m*-Control and *m*-A2-165 (designated as *m*ʹ-Control and *m*ʹ-A2-165, respectively) (Fig. 1H). This finding underscores the enhanced transgenerational impact of these *Faecalibacterium* pathobionts on offspring than their maternal counterparts.

### Effects of Faecalibacterium strains and a low-fiber diet on female mice

**Fig. 2:**
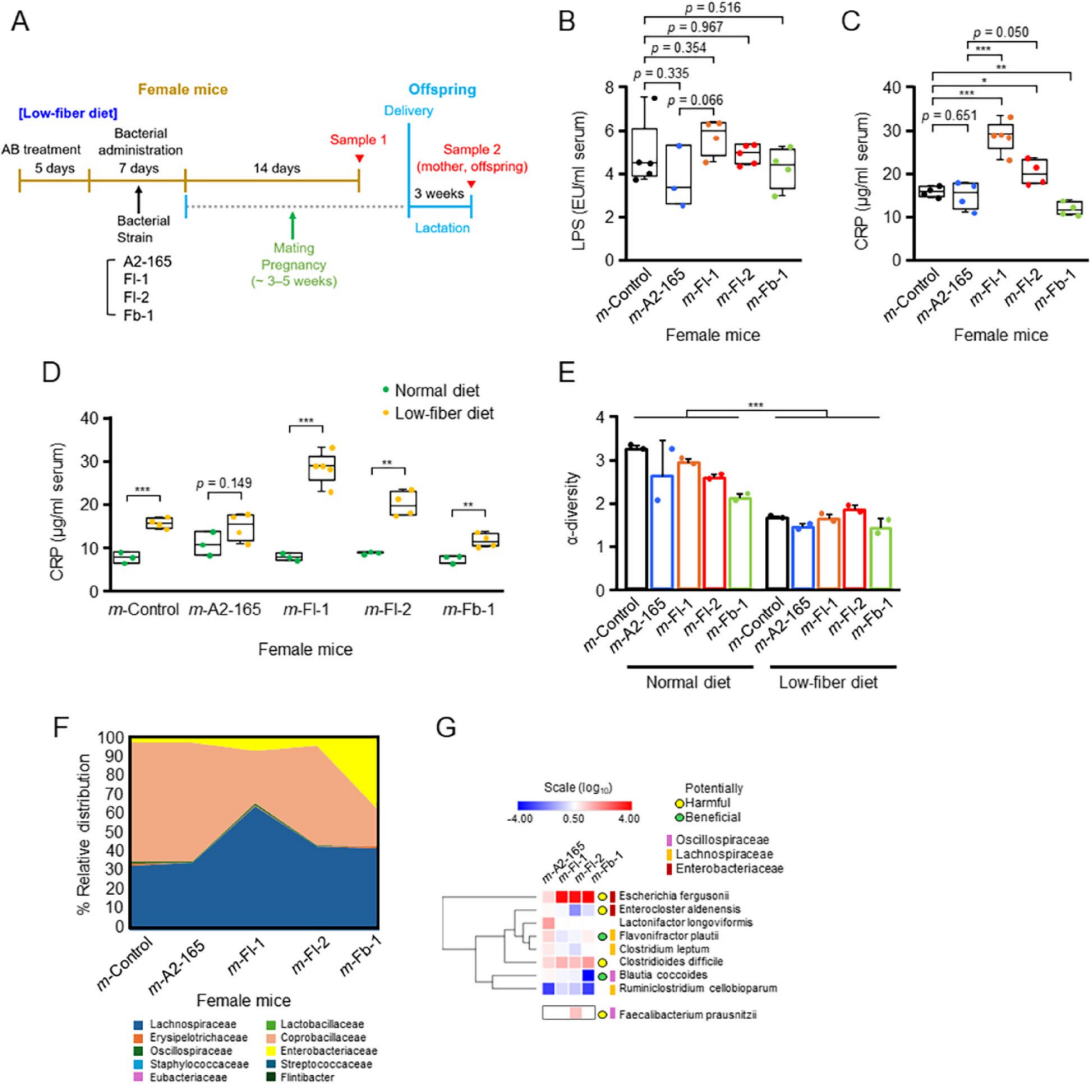
Effects of *Faecalibacterium* strains and a low-fiber diet on female mice. **A** Mouse model used for testing *Faecalibacterium* strains. Sample 1 was collected from female mice that did not undergo pregnancy, whereas sample 2 was obtained from mother mice and offspring just before weaning. **B** Serum LPS levels in female mice (*n* = 5 per group). **C** Serum C-reactive protein (CRP) levels in female mice (*n* = 5 per group). **D** Side-by-side comparison of serum CRP levels in female mice fed a normal diet versus those fed a low-fiber diet. **E** α-Diversity values in female mice on a low-fiber diet compared to those in female mice on a normal diet (Fig. 1F). **F** Gut microbiota profiles of the female mice. **G** Heatmap of hierarchical clustering displaying the relative abundance of key bacterial species among the mouse groups on day 14 post-administration. The log_10_ ratios of bacterial species in *m-*A2-165, *m-*Fl-1, *m-*Fl-2, or *m-*Fb-1 relative to *m-*Control are displayed. Potentially harmful or beneficial bacteria, along with the three key families to which they belong, are color-coded. Students *t*-test; *p < 0.05; **p < 0.01; ***p < 0.001. Statistics are presented for results that met the specified *p*-value thresholds and for others that were noteworthy

Although we observed the effects of *Faecalibacterium* strains on female mice and their offspring (Fig. 1), we hypothesized that these effects would be more pronounced if female mice were on a low-fiber diet. This hypothesis is based on the documented consequences of the global trend of inadequate fiber intake over recent decades, which has significantly influenced the human gut microbiome as a major contributing factor [15, 40]. Hence, we formulated a customized low-fiber diet containing approximately 8% fermentable fiber, compared to approximately 20% in a normal chow diet. In sample 1, collected on day 14 post-administration (Fig. 2A), the low-fiber diet did not significantly increase overall or strain-specific serum LPS levels in female mice (Fig. 2B); these levels were comparable to those observed in mice fed a normal chow diet (Fig. 1D). Notably, under both dietary conditions, the *m*-Fl-1 group exhibited a moderate increase in LPS levels compared to *m*-Control (Fig. 1D) and *m*-A2-165 (Fig. 2B). In contrast, the low-fiber diet significantly elevated serum CRP levels in the *m*-Fl-1 and *m*-Fl-2 groups—particularly in *m*-Fl-1—indicating increased systemic inflammation (Fig. 2C). In side-by-side comparisons, CRP levels were elevated in all groups on the low-fiber diet relative to those on the normal diet, with the *m*-A2-165 group exhibiting the smallest increase, potentially reflecting the beneficial effects of the administered strain (Fig. 2D). Reduced fiber intake resulted in a significant decrease in overall cecal SCFA levels. Among the SCFAs, only butyrate was reliably measured in the low-fiber diet groups, with levels remaining below 4 μmol/g of cecal content across all groups (Additional file 1: Fig. S2A). This contrasts sharply with the results shown in Fig. 1E, where *m*-A2-165 exhibited butyrate levels more than three times higher. Similarly, no significant differences in α-diversity were detected among the cecal microbiota of the different mouse groups, consistent with findings from the normal chow diet (Fig. 2E). However, a significant reduction in α-diversity was observed when comparing mice on a normal diet to those on a low-fiber diet, with the latter demonstrating lower values (Fig. 2E). Significant global-level changes in the microbiota structure were observed across all mouse groups, including *m*-Control, compared to those on a normal diet (Fig. 2F). The microbiota structure of *m*-A2-165 closely resembled that of the *m*-Control, whereas other groups exhibited more significant alterations, including increased proportions of the Enterobacteriaceae family, suggesting more severe dysbiosis (Fig. 2F). At the species level, significant increases in the prominent pathogens *E. fergusonii* [38] and *C. difficile* [41] were observed in the *m*-Fl-1, *m*-Fl-2, and *m*-Fb-1 groups compared to those in the *m*-A2-165 group (Fig. 2G). Additionally, lower levels of potentially beneficial species, such as *Flavonifractor plautii* [42] and *B. coccoides* [35], were observed in the *m*-Fl-1, *m*-Fl-2, or *m*-Fb-1 groups compared to those in the *m*-A2-165 group (Fig. 2G). Although no *Faecalibacterium* strains were detected using DNA sequencing in female mice on a normal diet, at least one strain (Fl-2) was detected in this group (Fig. 2G).

### Severe impact of Faecalibacterium strains and a low-fiber diet on the offspring

**Fig. 3:**
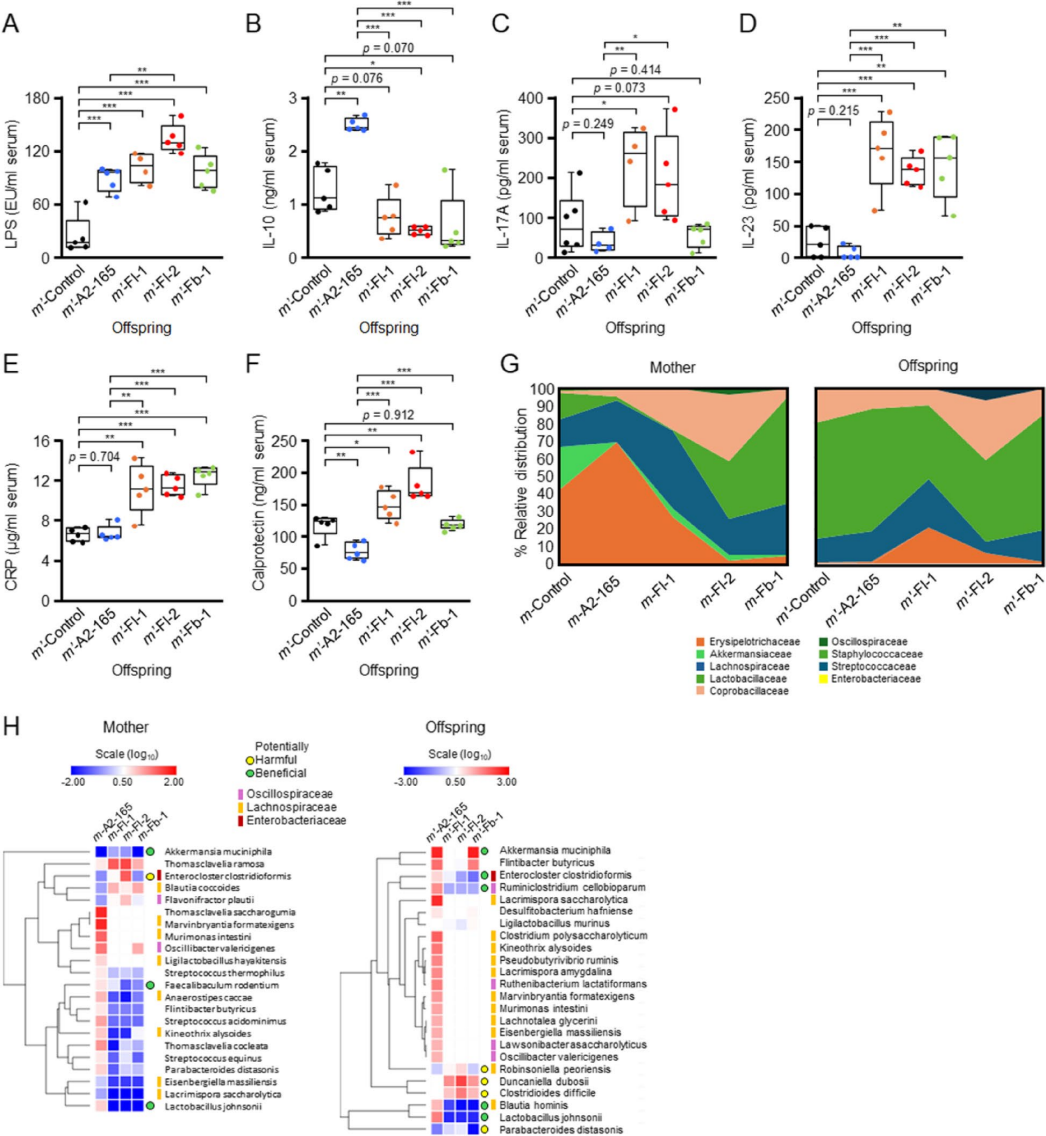
Severe impact of *Faecalibacterium* strains and a low-fiber diet on the offspring. **A** Serum LPS levels in offspring. **B** Serum interleukin (IL)−10 levels in offspring. **C** Serum IL-17A levels in offspring. **D** Serum IL-23 levels in offspring. **E** Serum C-reactive protein (CRP) levels in offspring. **F** Serum calprotectin levels in offspring. **G.** Gut microbiota profiles of mother mice and their offspring. **H** Heatmap of hierarchical clustering showing the relative abundance of key bacterial species among the mother and offspring groups. For the serum assays, 5 mice per group, each originating from a different mother mouse, were used. The log_10_ ratios of bacterial species in *m-*A2-165, *m-*Fl-1, *m-*Fl-2, or *m-*Fb-1 relative to those in *m-*Control or their corresponding offspring are presented. Potentially harmful or beneficial bacteria, along with three key families to which the bacterial species belong, are color-coded. Students *t*-test; *p < 0.05; **p < 0.01; ***p < 0.001. Statistics are presented for results that met the specified *p*-value thresholds and for others that were noteworthy

Although female mice on a low-fiber diet showed an increased impact of *Faecalibacterium* pathobionts, this effect was more pronounced in their offspring. All offspring groups displayed significantly elevated serum LPS levels compared to those of mothers on a normal diet (Fig. 3A). Furthermore, all offspring groups from *Faecalibacterium*-administered mothers had higher LPS levels than the *m*ʹ-Control group, with *m*ʹ-Fl-2 exhibiting the highest levels (Fig. 3A). Although *m*ʹ-A2-165 showed increased serum LPS levels, it also exhibited significantly elevated serum levels of the anti-inflammatory cytokine IL-10 (Fig. 3B). By contrast, IL-10 levels in the *m*ʹ-Fl-1, *m*ʹ-Fl-2, and *m*ʹ-Fb-1 groups were significantly lower than those in the *m*ʹ-Control group (Fig. 3B). Additionally, *m*ʹ-A2-165 displayed an inverse pattern of IL-17A (Fig. 3C), IL-23 (Fig. 3D), CRP (Fig. 3E), and calprotectin (Fig. 3F) levels compared to *m*ʹ-Fl-1 and *m*ʹ-Fl-2, which showed significantly elevated levels of these markers. *m*ʹ-Fb-1 also exhibited significantly increased serum IL-23 (Fig. 3D) and CRP (Fig. 3E) levels, but not IL-17A or calprotectin (Fig. 3C and F). Thus, the effect of *Faecalibacterium* was significantly more pronounced in the offspring, which is notable considering that female mice were directly treated. By contrast, no significant differences in cecal SCFA levels were observed among the offspring groups (Additional file 1: Fig. S2B). The overall cecal microbiota structure of the offspring resembled that of their mothers, particularly in the pairs of strains Fl-2 and Fb-1 (Fig. 3G). The gut microbiota structures of the mothers differed from those of nonreproductive female mice (sample 1) (Fig. 2E), suggesting that pregnancy, delivery, and lactation may have influenced microbiota composition. The human gut microbiome undergoes significant changes during pregnancy [43], including a decrease in SCFA producers [43], a pattern observed in the mothers’ gut microbiota (Fig. 3G). Similar to the observations in female mice from sample 1, species from the families Lachnospiraceae and Oscillospiraceae were the most differentially affected across the mouse groups (Fig. 3H). Marginal changes in potentially beneficial species in *m*-Fl-1, *m*-Fl-2, or *m*-Fb-1 compared to *m*-A2-165 included *Akkermansia muciniphila* [44], *Faecalibaculum rodentium* [45], and *Lactobacillus johnsonii* [46] (Fig. 3H). Increase in potentially harmful species in *m*-Fl-1, *m*-Fl-2, or *m*-Fb-1 compared to *m*-A2-165 included *Enterocloster clostridioformis* [47] (Fig. 1G). Although *E. clostridioformis* is typically considered beneficial, it can act as an opportunistic pathogen under certain conditions, and is associated with bloodstream infections [47]. In the offspring, potentially beneficial species decreased in *m*-Fl-1, *m*-Fl-2, or *m*-Fb-1 compared to *m*-A2-165 included *A. muciniphila* [44], *E. clostridioformis* [48], *Ruminoclostridium cellobioparum* [49], *B. hominis* [35], and *L. johnsonii* [46] (Fig. 3H). Potentially harmful species with an increased prevalence in *m*-Fl-1, *m*-Fl-2, or *m*-Fb-1 compared with *m*-A2-165 included *Robinsoniella peoriensis* [50], *Duncaniella dubosii* [51], and *C. difficile* [41] (Fig. 3H).

### Histological analysis indicates compromised intestinal barriers in affected offspring

**Fig. 4:**
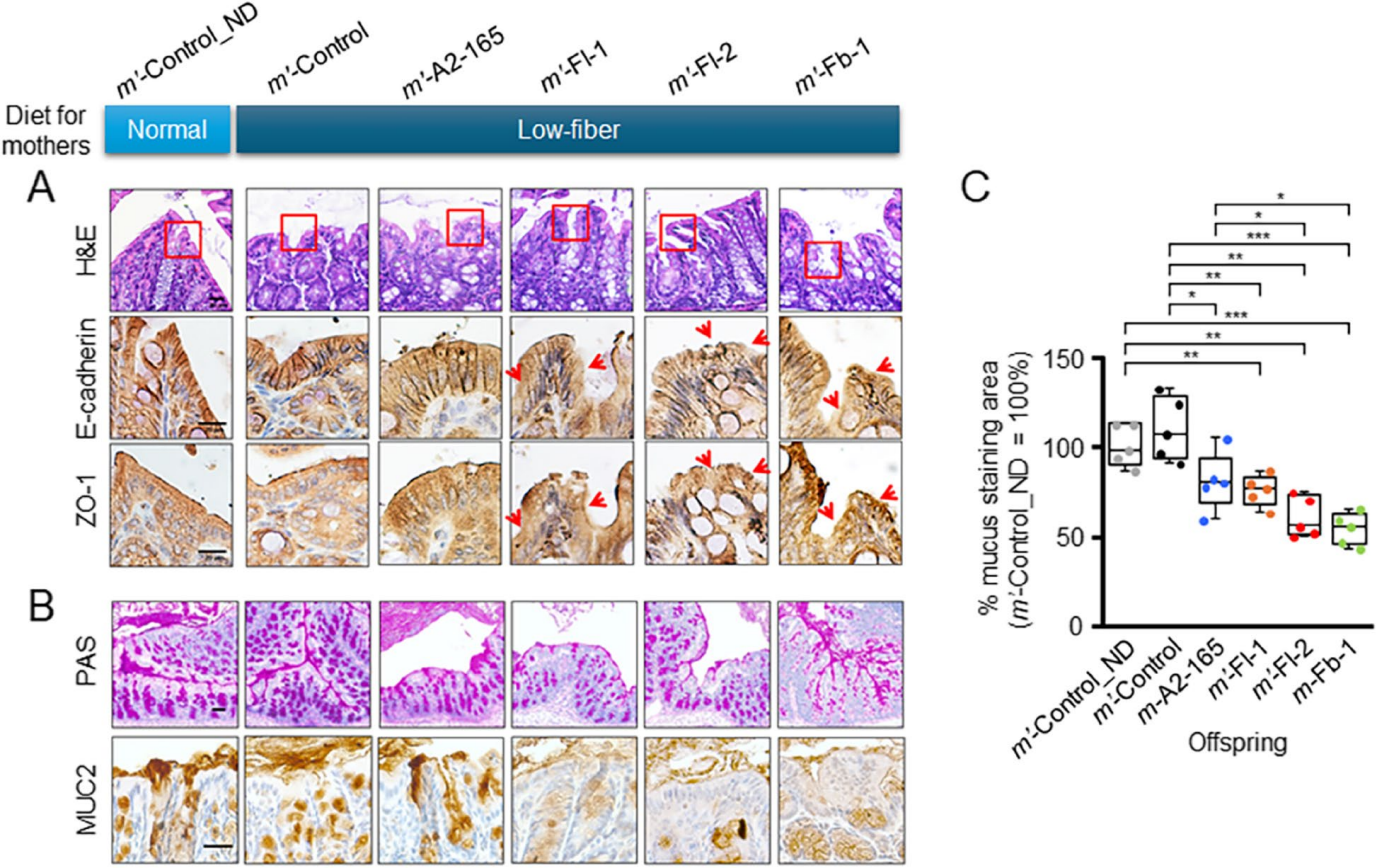
Histological analysis suggests compromised intestinal barriers in affected offspring. **A** Histological and immunohistochemical analyses of E-cadherin and zonula occludens-1 (ZO-1) in the large intestinal epithelium. Red boxes in the hematoxylin and eosin (H&E)-stained sections mark areas magnified in the immunohistochemical staining panels for E-cadherin and ZO-1. Regions with reduced staining intensity for E-cadherin or ZO-1, as well as epithelial disruption, are indicated by red arrowheads, particularly in the *m*ʹ-Fl-1, *m*ʹ-Fl-2, and *m*ʹ-Fb-1 groups. Scale bar = 20 µm.** B** Periodic acid-Schiff (PAS) staining and immunohistochemical staining for MUC2 in the large intestinal mucosa. PAS staining, which marks mucin present beneath and above the epithelium in pink, appeared less intense and thinner in *m*ʹ-Fl-1, *m*ʹ-Fl-2, and *m*ʹ-Fb-1 compared to *m*ʹ-A2-165 and the two control groups. Similarly, MUC2 immunohistochemical staining showed a reduced mucin layer thickness in the same groups. Scale bar = 20 µm. **C** Relative mucus production across mouse groups. PAS-positive areas in histological images of the large intestine were quantified as a percentage of the total mucosal area within defined fields at 400 × magnification. Three randomly selected fields per image were analyzed using ImageJ. Measurements were normalized to the *m*ʹ-Control group and expressed as percentage values. Students *t*-test; *p < 0.05; **p < 0.01; ***p < 0.001

As offspring of mice on a low-fiber diet and administered pathobionts exhibited increased systemic inflammation and altered gut microbiota (Fig. 3), we histologically analyzed the large intestine tissues to assess potential damage. When evaluating the tight junction protein zonula occludens-1 (ZO-1) and the adherens junction protein epithelial cadherin (E-cadherin) in formalin-fixed tissues, no significant differences were observed between the *m*ʹ-A2-165 group and the control groups *m*ʹ-Control and *m*ʹ-Control_ND (fed a normal chow diet) (Fig. 4A). By contrast, offspring of female mice treated with strains Fl-1, Fl-2, or Fb-1 displayed significantly reduced ZO-1 and E-cadherin levels, with the most pronounced reductions observed in the *m*ʹ-Fl-2 group (Fig. 4A). To further investigate the barrier integrity, we assessed the mucus layer thickness using periodic acid–Schiff/alcian blue (PAS/AB) staining and antibody-mediated MUC2 staining. Both methods consistently showed that the *m*ʹ-Fl-1, *m*ʹ-Fl-2, and *m*ʹ-Fb-1 groups exhibited significantly thinner mucus layers and reduced MUC2 production in goblet cells compared to the control groups and the *m*ʹ-A2-165 group, as observed in histological analysis (Fig. 4B) and confirmed by quantitative measurements (Fig. 4C).

### Severe hair loss in affected offspring

**Fig. 5:**
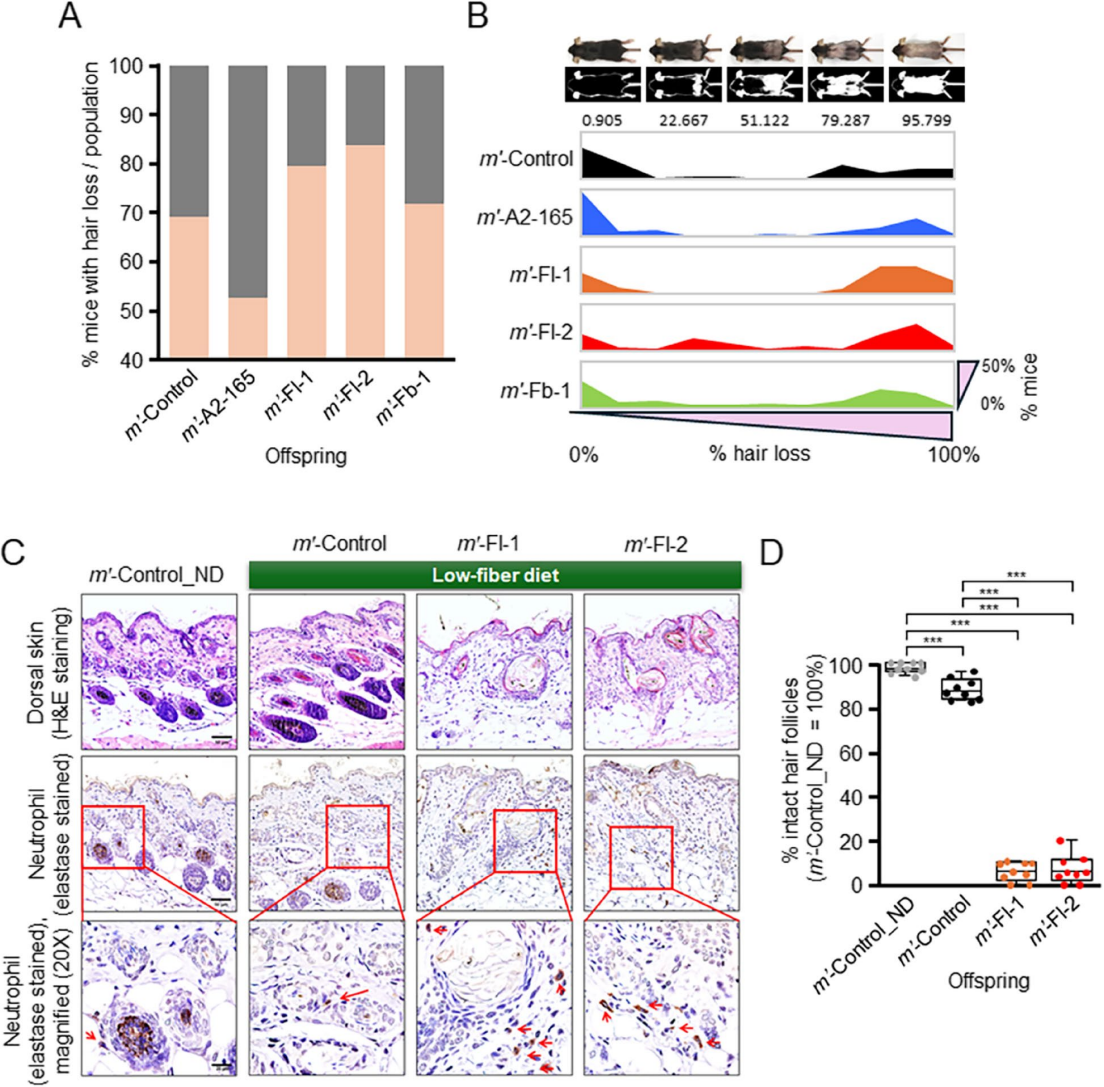
Offspring of mothers fed a low-fiber diet may exhibit severe hair loss and inflamed skin tissue. **A** Percentage of mice exhibiting hair loss in each group. Hair loss was defined as affecting at least 5% of fur-covered areas (*n* = 55–67 per group, derived from 11 mother mice). **B** Severity of hair loss at the individual level. Proportion of affected areas are shown for each mouse group (*n* = 55–67 per group, derived from 11 mother mice). **C** Histological comparison of affected dorsal skin in offspring versus control mice. Hematoxylin and eosin (H&E)-stained sections illustrate the findings. The skin of offspring from female mice fed a normal chow diet without bacterial administration (*m*ʹ-Control_ND) exhibits healthy skin with intact hair follicles. By contrast, the unaffected skin of offspring from females on a low-fiber diet without bacterial administration (*m*ʹ-Control) shows slightly increased defective hair follicles. The *m*ʹ-Fl-1 and *m*ʹ-Fl-2 groups exhibit a significantly reduced number of intact hair follicles and increased neutrophil infiltration, particularly around defective follicles. **D** Relative intact hair follicles across mouse groups. The percentage of intact hair follicles per defined field was quantified from histological images of skin tissues (3 mice per group, 3 fields per mouse, 200 × magnification). Students *t*-test; *p < 0.05; **p < 0.01; ***p < 0.001

A prominent consequence of elevated systemic inflammation in the offspring was severe hair loss, which began approximately 2 weeks after birth following an initial phase of normal fur growth. This condition persisted for approximately 1 week during the lactation period and gradually improved as weaning commenced (Fig. 5A and B; Additional file 1: Fig. S3). The consistent and widespread distribution of hair loss across the dorsal skin (Additional file 1: Fig. S3)—rather than in patchy regions—further supports the interpretation that this condition was of systemic pathological origin, rather than a manifestation of normal hair cycle variation. Interestingly, hair loss occurred even in the absence of *Faecalibacterium* administration when the female mice were maintained on a low-fiber diet, as observed in the *m*ʹ-Control group (Fig. 5A). Approximately 69% of the *m*ʹ-Control mice exhibited hair loss, defined as a positive case when at least 5% of the surface area with fur was affected (Fig. 5A). The *m*ʹ-Fl-1 and *m*ʹ-Fl-2 groups displayed more extensive hair loss than the *m*ʹ-Control group, with incidence rates reaching approximately 76% and 84%, respectively (Fig. 5A). By contrast, the *m*ʹ-A2-165 group showed reduced hair loss than the *m*ʹ-Control group (~ 53%), whereas the *m*ʹ-Fb-1 group exhibited hair loss at levels comparable to those of the *m*ʹ-Control group (Fig. 5A). On an individual level, the *m*ʹ-Fl-1 and *m*ʹ-Fl-2 groups also displayed the most severe hair loss patterns, with the highest proportions of mice exhibiting advanced hair loss compared to those in *m*ʹ-A2-165, *m*ʹ-Fb-1, and *m*ʹ-Control groups (Fig. 5B).

### Histological analysis of affected dorsal skin tissues

Histological analysis of dorsal skin tissues from the *m*ʹ-Fl-1 and *m*ʹ-Fl-2 groups with hair loss revealed significantly fewer hair follicles compared to the normal skin tissues of the *m*ʹ-Control_ND group (offspring of a normal chow-fed mother without bacterial administration), which did not exhibit hair loss, or the unaffected skin tissues of the *m*ʹ-Control group (offspring of a low fiber-fed mother without bacterial administration) (Fig. 5C). Quantification of intact follicles in histological images supported the visual observations, confirming that follicular integrity was significantly compromised in these groups (Fig. 5D). Elastase staining revealed that the affected skin tissues from the *m*ʹ-Fl-1 and *m*ʹ-Fl-2 groups exhibited increased neutrophil recruitment, suggesting heightened inflammation (Fig. 5C). However, these tissues did not demonstrate significantly reduced adherence junction protein E-cadherin or tight junction proteins claudin-1 and claudin-7 in the epithelium or around hair follicles, which are hallmarks of a compromised epithelial barrier. This observation contrasts with the many skin lesions typically associated with AD [8, 52, 53]. The mild nature of these changes aligns with the temporary and reversible nature of the abnormalities in the affected skin tissues, which resolve after weaning.

## Discussion

In this study, we investigated the role of *Faecalibacterium* pathobionts in systemic conditions, with a specific focus on AD, building on previous research that identified significant enrichment of these bacteria in young patients with AD [24]. By administering female mice strains of *F. longum* and *F. butyricigenerans*, along with *F. duncaniae* A2-165 as a beneficial control strain [19], we demonstrated that *Faecalibacterium* pathobionts, particularly *F. longum* strains, and to a lesser extent, the *F. butyricigenerans* strain, influenced the gut microbiome and systemic health of female mice, with even more pronounced effects observed in their offspring. Our findings provide compelling evidence that maternal *Faecalibacterium* pathobionts play a key role in the development of systemic conditions in the offspring.

Although the effects of the administered bacteria on female mice and their offspring were mild when on a normal diet, these effects became significantly more severe when the female mice were fed a low-fiber diet. This observation was evident from the increase in factors linked to systemic inflammation, alterations in gut microbiota, disruption of gut mucosal integrity, and severe hair loss accompanied by localized skin inflammation in the offspring. This exacerbation is partly attributed to the reduced production of SCFAs, particularly butyrate, which plays a critical role in maintaining gut homeostasis [23, 54]. The exacerbation of systemic symptoms caused by a low-fiber diet mirrors contemporary dietary trends, highlighting the critical role of modern dietary trends in the development of AD and other chronic conditions. A low-fiber diet, providing approximately 40% of normal fiber intake, was sufficient to mitigate the negative effects of *Faecalibacterium* pathobionts on offspring health. This synergistic relationship between maternal diet and gut microbiota dysbiosis underscores the need for a holistic approach to address chronic conditions such as AD. Previous studies have demonstrated a strong link between fiber intake, gut microbiome diversity, and overall health [55–57], indicating that modern dietary patterns may significantly contribute to the increasing prevalence of chronic conditions in human populations.

Several lines of evidence support the key features of AD that were observed in our mouse models. In the offspring of female mice administered pathobionts, we observed significantly elevated LPS, IL-17A, IL-23, CRP, and calprotectin levels, along with decreased IL-10 levels. Increasing evidence suggests that IL-17 and IL-23 play critical roles in the pathogenesis of AD and psoriasis [58–60]. Additionally, CRP, previously implicated in systemic inflammation [61], has recently been linked to AD [62], whereas calprotectin is a well-established biomarker for intestinal inflammation and related diseases [63]. *Faecalibacterium* pathobionts exhibited a diminished capacity to reduce the anti-inflammatory cytokine IL-10 compared to the beneficial strain A2-165, as demonstrated in PBMCs. Moreover, the skin of the offspring exhibited significant abnormalities including defective hair follicles and increased neutrophil recruitment. Despite compelling evidence linking maternal *Faecalibacterium* pathobionts to their potential roles in AD, this study was limited by its reliance on a nonhuman model. For instance, additional skin impairments such as epithelial barrier dysfunction were not observed. This observation is in contrast to many allergic inflammatory diseases, including AD, which typically involve both inflammation and epithelial barrier disruption [8, 52, 53]. Furthermore, unlike human AD that is characterized by severe itching driven by systemic type 2 immune responses [3, 5, 13], the inflamed skin in this mouse model did not exhibit noticeable itchiness. Thus, our model may represent only a mild form of AD and may not fully replicate the complexities of conditions observed in humans. This limitation may partly stem from the intrinsic incompatibility between mice and bacteria of human origin, which do not naturally colonize the murine gut. This observation is supported by the high levels of colonization resistance in mice [64]. Consistently, the administered *Faecalibacterium* strains were undetectable in female mice at 14 days post-administration, except for one strain, Fl-2, which persisted on a low-fiber diet. Similarly, none of these strains was detected in the offspring. This finding contrasts with those in young patients with AD, where *Faecalibacterium* pathobionts are enriched [24]. Hence, the symptoms observed in the offspring are probably attributable to vertically transmitted aberrant microbiota. Hair loss, which is one of the primary symptoms, resolved after weaning. This observation is consistent with that in humans that the rapid maturation of the gut microbiome begins with the cessation of the lactation period [65].

**Fig. 6:**
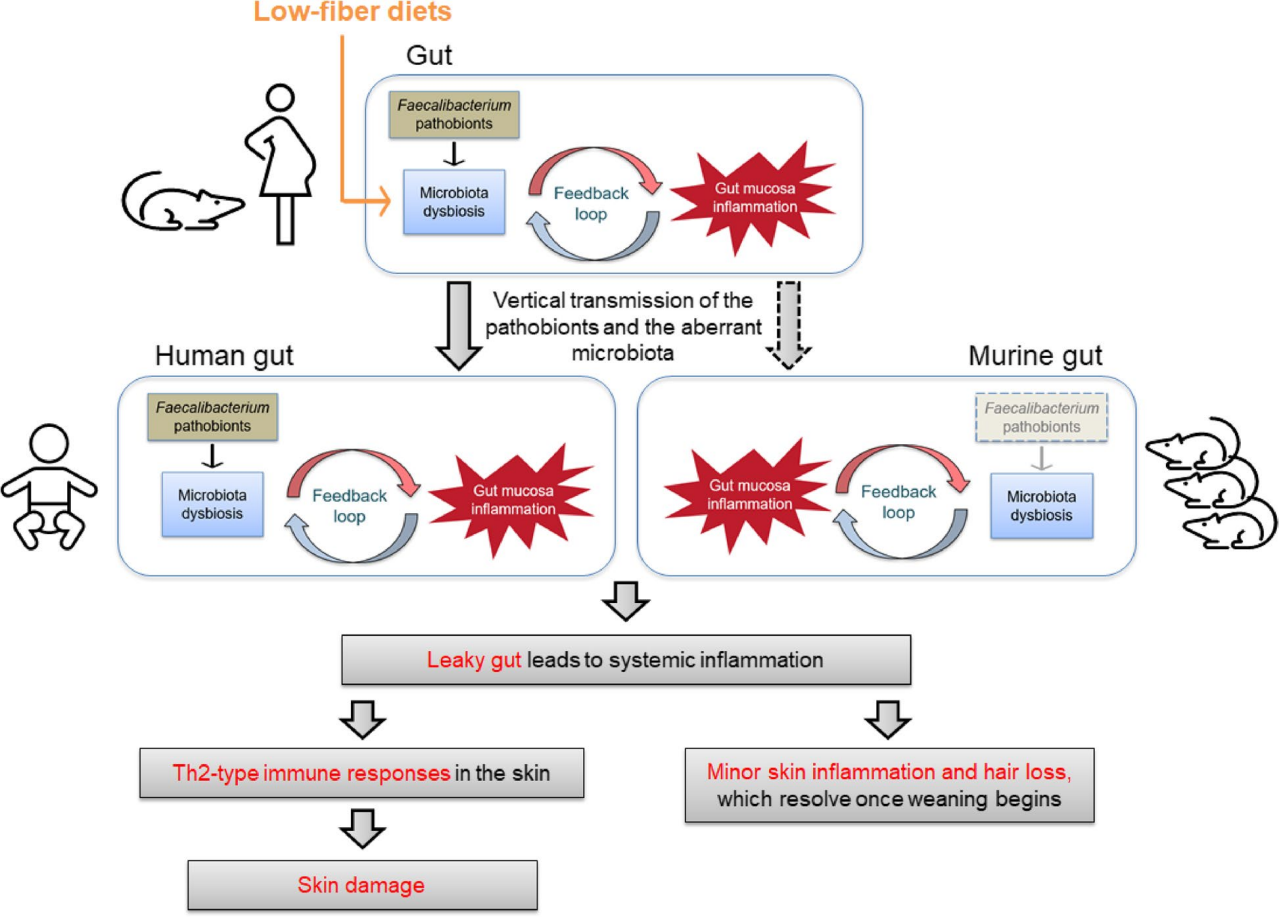
Updated model for the development of atopic dermatitis and related systemic conditions highlights key mechanisms influenced by gut microbiota. In females, an increase in *Faecalibacterium* pathobionts (e.g., strains of *F. longum* and *F. butyricigenerans*) relative to beneficial counterparts (e.g., strains of *F. duncaniae* strains) may drive gut mucosal inflammation. This inflammation, in turn, could promote the growth of pathobionts, creating a positive feedback loop, as suggested in a previous study [24]. This loop is exacerbated by low-fiber diets, partly due to reduced production of short-chain fatty acids (SCFAs), particularly butyrate. *Faecalibacterium* pathobionts and the associated aberrant microbiota composition can be vertically transmitted to offspring [24], perpetuating a similar feedback loop characterized by dysregulated mucosal inflammation. Compromised gut permeability arising from chronic inflammation may lead to systemic-level inflammation. In human infants, this systemic inflammation may manifest as skin damage through aberrant T_H_2-type immune responses in the skin [7, 8]. By contrast, mice with high colonization resistance to nonnative gut bacteria^67,68^ show limited colonization and vertical transmission of human-origin *Faecalibacterium* strains. Consequently, these mice exhibit only mild AD-like symptoms without the complexities observed in humans. The offspring display mild skin inflammation and localized hair loss in affected areas, which resolves after weaning begins

The induction of gut microbiome dysbiosis in female mice by *Faecalibacterium* pathobionts and their subsequent effects on offspring provide new insights into the mechanisms underlying AD and related systemic conditions. These findings support our previously proposed model of AD development, highlighting an imbalance between pathobionts and their beneficial counterparts within the *Faecalibacterium* group as a key causal factor [24]. This imbalance may establish a positive feedback loop with mucosal inflammation, ultimately driving the onset and progression of AD [24]. A key factor contributing to this imbalance may be the higher tolerance of *F. longum* strains (i.e., L2-6-like strains) to oxidative stress than that of the beneficial *F. duncaniae* strains, such as A2-165 [25]. This study demonstrated the vertical transmission of aberrant maternal gut microbiota to offspring, shaping their gut microbiota composition and influencing their overall health. Building on these findings, we propose an updated model in which a positive feedback loop between *Faecalibacterium*-mediated gut microbiota dysbiosis and mucosal inflammation in the mother, which is further exacerbated by a low-fiber diet, drives systemic conditions in the offspring, such as AD, through vertical transmission (Fig. 6). The pathology of IBD shares significant similarities with this model of AD, including a comparable feedback loop between the gut microbiota and mucosal inflammation [66, 67], as well as similar patterns of vertical transmission [68, 69]. Consistently, *F. longum* strains (i.e., L2-6-like strains) were dominant in pediatric mucosal samples, and only these strains showed high intra-species abundance in Crohn’s disease [25]. A prospective cohort study on Crohn’s disease identified predictive gut bacteria for the disease, including members of the Oscillospiraceae family, to which *Faecalibacterium* pathobionts belong [70]. Furthermore, a recent preliminary study has reported that children on the autism spectrum have higher *Faecalibacterium* levels [71]. By contrast, a growing body of research has linked reduced levels of beneficial *Faecalibacterium* to various diseases, including Crohn’s disease [19], ulcerative colitis [20], irritable bowel syndrome [72], metabolic conditions [21], neurological conditions [73], and cancer [74]. These findings underscore the adverse consequences of reduced beneficial and crucial functions of *Faecalibacterium* in maintaining physiological integrity. However, this study highlights the need to explore the potential contributions of pathobiont counterparts to the development of these diseases.

## Conclusion

The findings from this study provide compelling evidence that maternal *Faecalibacterium* pathobionts play a critical role in the development of systemic conditions in offspring, offering valuable insights into the etiology of AD. Identifying and potentially targeting pathogenic strains of *Faecalibacterium* could be pivotal for achieving a precise diagnosis and treatment of AD and related systemic conditions. Notably, administration of the beneficial *F. duncaniae* strain A2-165 to female mice reduced systemic inflammation and hair loss in their offspring. This observation suggests potential therapeutic strategies to restore maternal gut health and improve AD outcomes in the offspring. These findings underscore the potential for developing effective live biotherapeutic products [75] based on beneficial *Faecalibacterium* strains. Additionally, implementing dietary modifications as adjunct therapies for mothers could serve as a novel approach for preventing AD in offspring, addressing this increasingly prevalent condition.

Future studies should explore the step-by-step mechanisms by which *Faecalibacterium* pathobionts induce gut microbiota dysbiosis and facilitate the vertical transmission of aberrant microbiota, ultimately affecting offspring health. Additionally, the factors regulating the balance between beneficial and harmful *Faecalibacterium* strains need to be identified to advance our understanding of the mechanisms underlying AD. This insight could lead to improved diagnostic and therapeutic strategies, help address the rising prevalence of AD, and improve the quality of life of affected individuals.

## Supplementary Information

Supplementary Material 1. Additional file 1: Fig. S1. Effects of *Faecalibacterium* strains on female mice. A. Serum CRP levels in groups of female mice. B. Serum calprotectin levels in groups of female mice. C. Serum IL-17A levels in groups of female mice.
Fig. S2. Cecal short-chain fatty acid (SCFA) levels in Female mice fed a low-fiber diet and their offspring. A. SCFA levels in female mice. Among the SCFAs, only butyrate was reliably measured. B. SCFA levels in offspring
Fig. S3. Offspring of mice administered *Faecalibacterium* strains and fed a low-fiber diet exhibit varying levels of hair loss. A comparison was made between 30 mice in each group

## Data Availability

The data analyzed and discussed in this study are available in the manuscript and online supplementary material. Sequencing data for each sample deposited in NCBI are available under BioProject accession numbers PRJNA1227222 (female), PRJNA1227227 (mother), and PRJNA1227231 (offspring). These accession numbers are not yet publicly available. For reviewers, we provide the following access links: PRJNA1227222 PRJNA1227227 PRJNA1227231
