# Supplementary material for: Maternal Faecalibacterium pathobionts and low-fiber diets synergize to impact offspring health: implications for atopic dermatitis
Source: Microbiome. 2025 Aug 29;13:192. doi: 10.1186/s40168-025-02194-8 (PMC12395903; doi:10.1186/s40168-025-02194-8)
Supplement: Supplementary file 2 — Supplementary Material 1. Additional file 1: Fig. S1. Effects of Faecalibacterium strains on female mice. A. Serum CRP levels in groups of female mice. B. Serum calprotectin levels in groups of female mice. C. Serum IL-17A levels in groups of female mice. Fig. S2. Cecal short-chain fatty acid (SCFA) levels in Female mice fed a low-fiber diet and their offspring. A. SCFA levels in female mice. Among the SCFAs, only butyrate was reliably measured. B. SCFA levels in offspring Fig. S3. Offspring of mice administered Faecalibacterium strains and fed a low-fiber diet exhibit varying levels of hair loss. A comparison was made between 30 mice in each group [file 40168_2025_2194_MOESM1_ESM.docx]

**Supplementary Information**

**
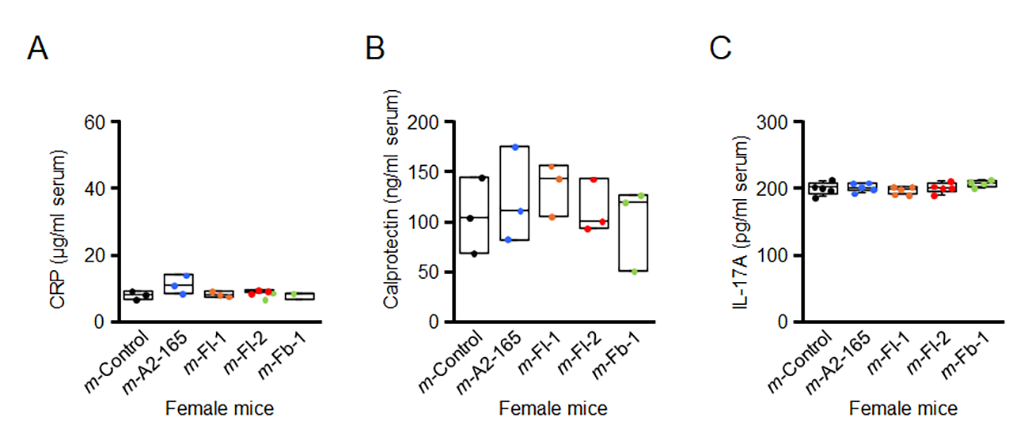
**

**Figure S1.** Effects of *Faecalibacterium* strains on female mice. **A.** Serum CRP levels in groups of female mice. **B.** Serum calprotectin levels in groups of female mice. **C.** Serum IL-17A levels in groups of female mice.

**
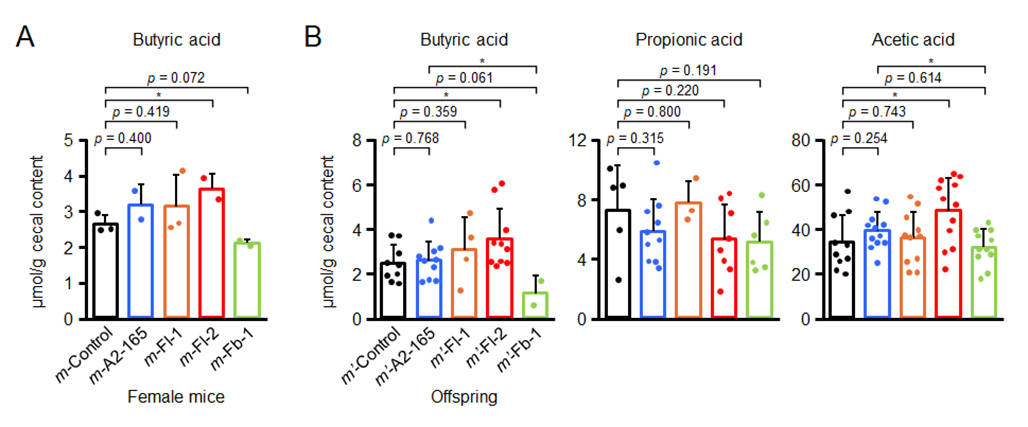
**

**Figure S2.** Cecal short-chain fatty acid (SCFA) levels in Female mice fed a low-fiber diet and their offspring. **A.** SCFA levels in female mice. Among the SCFAs, only butyrate was reliably measured. **B.** SCFA levels in offspring.

**
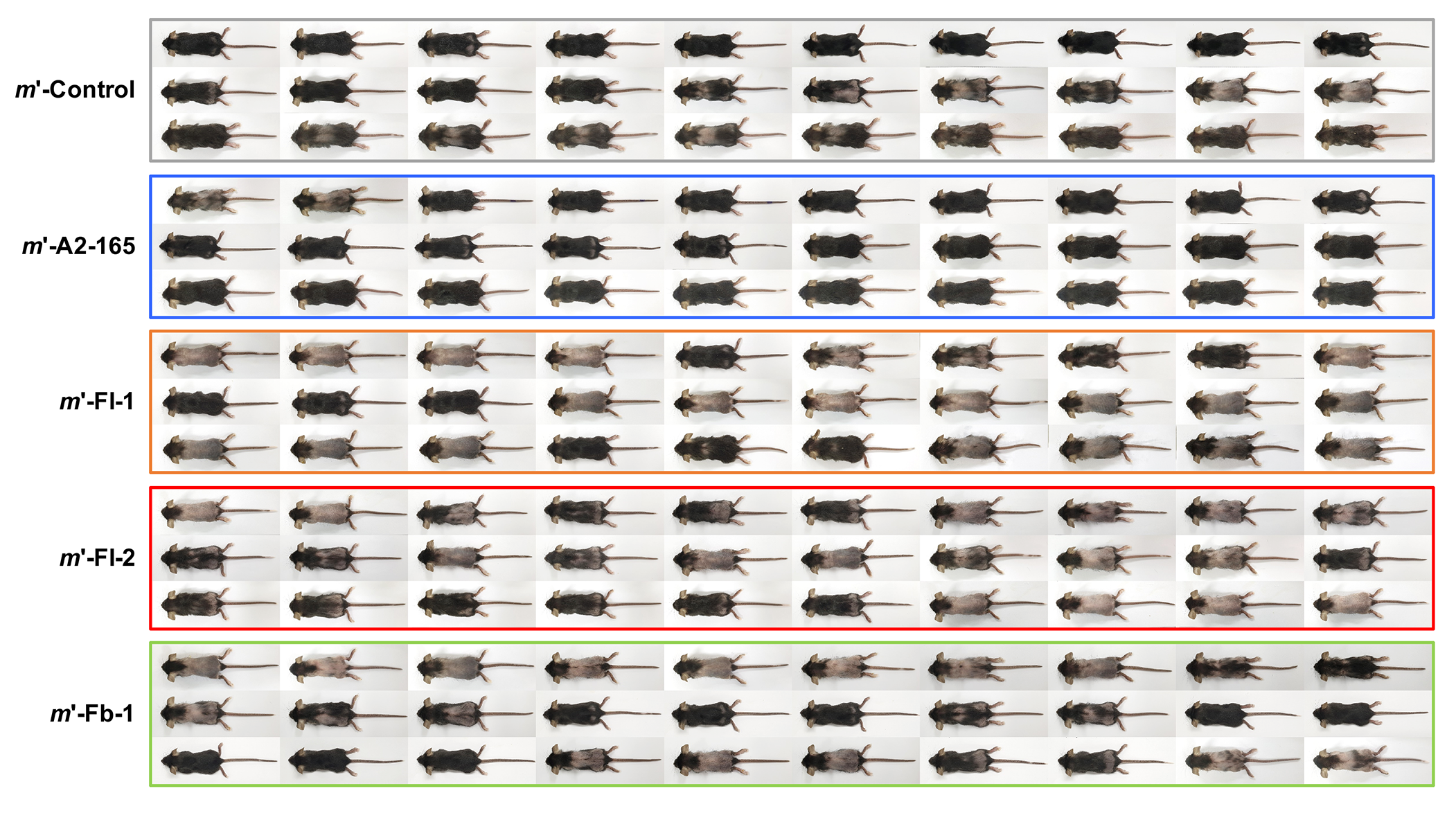
**

**Figure S3.** Offspring of mice administered *Faecalibacterium* strains and fed a low-fiber diet exhibit varying levels of hair loss. A comparison was made between 30 mice in each group.
